# Herpes Simplex Virus Diencephalitis Leading to Panhypopituitarism

**DOI:** 10.1210/jcemcr/luad050

**Published:** 2023-06-08

**Authors:** Caoimhe Casey, Antoinette O’Connor, Simon Cronin, Antoinette Tuthill

**Affiliations:** College of Medicine and Health, University College Cork, Cork, Ireland; Department of Endocrinology, Cork University Hospital, Cork, Ireland; Department of Neurology, Cork University Hospital, Cork, Ireland; Clinical Neurosciences, University College Cork, Cork, Ireland; College of Medicine and Health, University College Cork, Cork, Ireland; Department of Endocrinology, Cork University Hospital, Cork, Ireland

**Keywords:** panhypopituitarism, hypothalamus, HSV encephalitis, diabetes insipidus

## Abstract

Herpes simplex virus (HSV) is one of the most common causes of viral encephalitis. Hypothalamic-pituitary dysfunction has rarely been reported in HSV encephalitis, with few reports into the longer term outcomes for these patients. A 46-year-old male presented with a 10-day history of delirium, fever, and polydipsia. Initial computed tomography of the brain and cerebrospinal fluid cell counts were normal. Magnetic resonance imaging showed T2-hyperintensity affecting bilateral infundibuli, hypothalami, subthalamic nuclei, and optic radiations. Serial cerebrospinal fluid detected HSV1 DNA and we diagnosed him with HSV diencephalitis. He had marked biochemical abnormalities from the outset, with dramatic changes in serum sodium levels. He was ultimately diagnosed with permanent central diabetes insipidus and panhypopituitarism following evidence of central hypothyroidism, hypogonadotrophic hypogonadism, and a flat cortisol response to an insulin tolerance test. Neurocognitive recovery took several months, but subtle deficits in executive function and information processing remain. Hypothalamic hyperphagia developed as well as temperature dysregulation. He requires lifelong hormonal replacement and is undergoing regular endocrine follow up. This case highlights hypothalamic-pituitary dysfunction as a rare endocrine complication of HSV diencephalitis and illustrates the complexity of managing this in the long term.

## Introduction

Herpes simplex virus (HSV) is one of the most common causes of viral encephalitis [1]. Incidence in a large retrospective study was reported to be 2.2 per million population per year [2]. Clinical features include fever, headache, focal neurological signs, seizures, and altered consciousness. Hypothalamic-pituitary dysfunction has rarely been reported in HSV encephalitis (HSVE). Vesely et al reported a case of partial anterior hypopituitarism 27 years after initial HSVE presentation [3]. Ickenstein et al reported a case of herpes simplex encephalitis causing akinetic parkinsonism, panhypopituitarism, and central diabetes insipidus [4]. There are few reports into the long-term follow up and clinical outcomes of these patients.

We report a case of HSV diencephalitis leading to permanent panhypopituitarism and hypothalamic dysfunction. This case is 1 of the few reported cases of hypothalamic-pituitary dysfunction secondary to HSV encephalitis. We also uniquely illustrate the complexity of managing this disorder and its complications in the long term.

## Case Presentation

A 45-year-old lecturer presented to an outside hospital with a 10-day history of delirium, fever, and hallucinations. He had no medical history and was on no regular medications.

## Diagnostic Assessment

Initial laboratory investigations revealed hyponatremia (Table 1).

**Table 1.** Admission biochemistry

Biochemistry		Reference range	Alternative units
Sodium, mmol/L	**113**	132-144	113 mEq/L
Potassium, mmol/L	3.5	3.5-5.0	3.5 mEq/L
Chloride, mmol/L	81	95-107	81 mEq/L
Urea, mmol/L	2.6	2.5-7.0	15.62 mg/dL
Creatinine, μmol/L	77	64-104	0.87 mg/dL
Albumin, g/L	44	36-44	4.4 g/dL
Bilirubin, μmol/L	16	2-20	0.94 mg/dL
Alkaline phosphatase, µ/L	64	40-130	1.07 μkat/L
Alanine aminotransferase, µ/L	27	4-45	0.45 μkat/L
Aspartate aminotransferase, µ/L	32	6-42	0.53 μkat/L
Gamma-glutamyl transferase, µ/L	23	6-48	0.38 μkat/L
C-reactive protein, mg/L	9	0-10	0.9 mg/dL
Glucose, mmol/L	5.5		99 mg/dL
Serum osmolality, mOsm/kg	236	275-295	236 mmol/kg
Urine sodium, mmol/L	26		26 mEq/L
Urine osmolality, mOsm/kg	252		252 mmol/kg

The bold value refers to the pertinent abnormal result.

**Figure 1.**
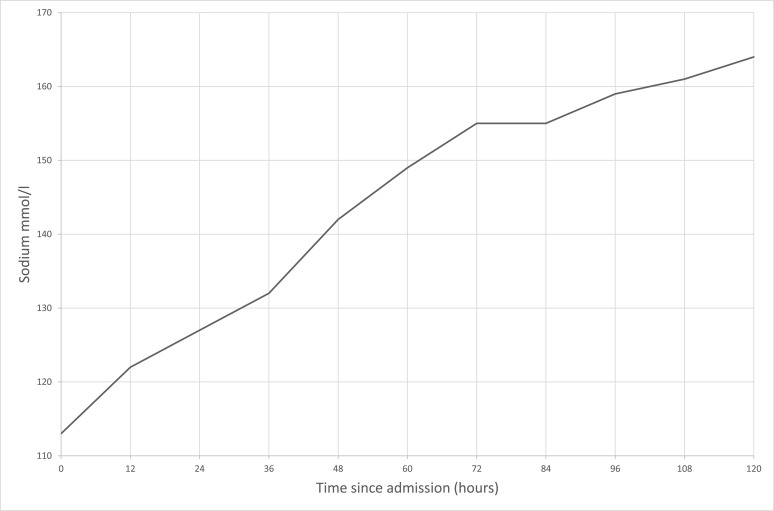
Sodium rise after admission.

A history of polydipsia was reported by the patient's wife, and fluid restriction was commenced for hyponatremia. Initial computed tomography of the brain was unremarkable. Cerebrospinal fluid analysis (CSF) on day 1 showed normal cell counts, but HSV polymerase chain reaction (PCR) was positive. Thyroid function tests showed a reduced free thyroxine of 9.7 pmol/L (reference range, 12-22) (0.75 ng/dL) and TSH 0.17 mIU/L (reference range, 0.4-3.8). Seventy-two hours later, sodium had dramatically risen to 155 mmol/L (155 mEq/L) (Fig. 1). He was transferred for further management to our acute tertiary referral hospital.

**Figure 2.**
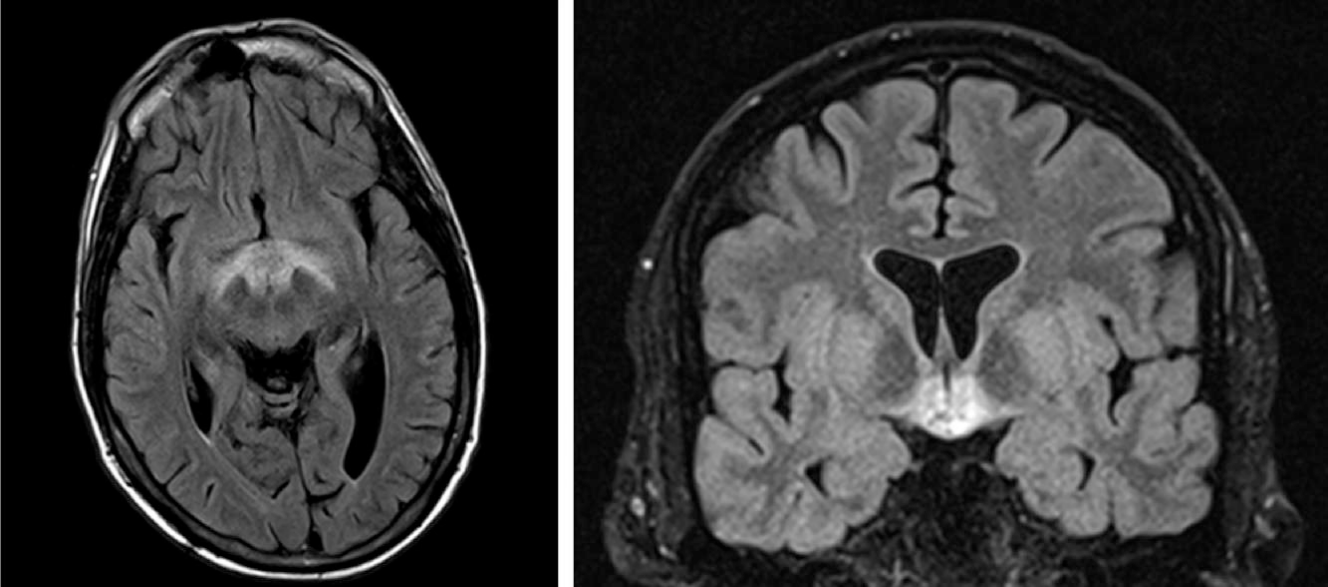
Magnetic resonance imaging of the brain 4 days into admission and 3 days after fluid restriction.

Magnetic resonance imaging of his brain showed symmetric T2-hyperintensity affecting bilateral infundibuli, hypothalami, subthalamic areas, and optic radiations, the “diencephalon” (Fig. 2). The temporal lobes and pons were unaffected.

## Treatment

He was treated with high-dose methylprednisolone 1 g once daily IV for 5 days and acyclovir 800 mg IV 3 times daily. Cortisol was not measured before commencing steroids. Seizures were treated with an antiepileptic agent. Repeat lumbar puncture on day 4 and day 17 showed lymphocytic pleocytosis, both again PCR-positive for HSV type 1. Repeat magnetic resonance imaging of the brain on day 14 showed progression of swelling to now also involve the bilateral insular cortex. Anti-NMDAR and LGI1 antibodies were negative. He was diagnosed with HSV diencephalitis. Sustained hyperpyrexia and progressive unconsciousness developed, requiring endotracheal ventilation.

**Figure 3.**
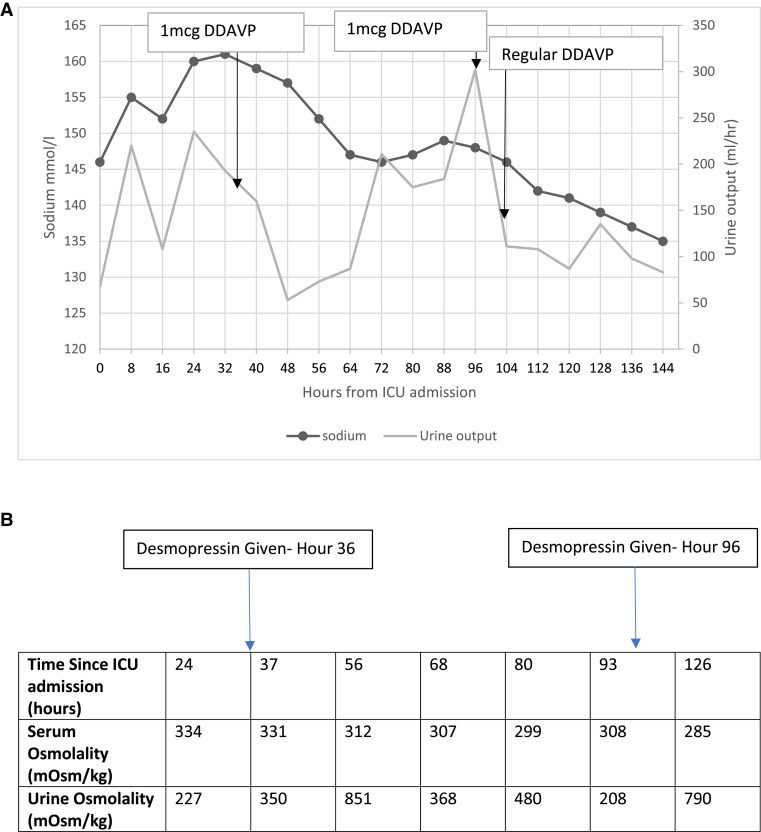
(A) Trend in sodium levels and urine output during intensive care unit (ICU) admission. (B) Trend in serum and urine osmolality during ICU admission.

The patient’s serum sodium level continued to rise, and polyuria was reported, although not accurately documented. At a sodium of 160 mmol/L(160 mEq/), serum osmolality was 334 mOsm/kg with a urine osmolality of 227 mOsm/kg and urine output of 235 mL/h, suggesting a diagnosis of diabetes insipidus. He was given 2 doses of desmopressin 1 mcg IV by the treating team. His sodium improved and urine output dropped to <60 mL/h. At this point, our endocrinology services became involved. Fluid replacement was given to correct the free water deficit. Regular desmopressin was commenced when polyuria persisted after several stat doses of desmopressin (Fig. 3). Hypernatremia slowly resolved with desmopressin treatment and the patient never became hyponatremic.

Anterior pituitary profile revealed hypogonadotrophic hypogonadism and central hypothyroidism (Table 2). On completion of 5 days of methylprednisolone, the patient was started on a reducing course of prednisolone and was ultimately switched to replacement dose hydrocortisone 10 mg twice daily because he was believed to have secondary adrenal insufficiency also given loss of other pituitary axes. Thyroxine replacement was started at 25 mcg/day 3 weeks into admission.

**Table 2.** Pituitary profile

		Reference range	Alternative units
TSH, mIU/L	0.10	0.4-3.8	0.10 mIU/L
Free thyroxine, pmol/L	6.9	12-22	0.54 ng/dL
LH, IU/L	0.2	1.7-8.6	0.2 mIU/mL
FSH, IU/L	1.1	1.5-12.4	1.1 mIU/mL
Testosterone, nmol/L	<0.4	10-28	<11.53 ng/dL
Prolactin, mU/L	257	86-324	12.08 μg/L

His inpatient course was also complicated by the development of bilateral pulmonary emboli, which were likely to be related to hypercoagulability associated with significant hypernatremia. Acyclovir was continued for 5 weeks until serial CSF testing was repeatedly HSV PCR negative.

Slow neurocognitive recovery followed for several months. Insulin tolerance testing 5 months after presentation revealed flat cortisol and GH response, indicating persistent panhypopituitarism (Table 3). Hydrocortisone was held for 24 hours before insulin tolerance testing according to our hospital protocol.

**Table 3.** Insulin tolerance test

	Glucose, mmol/L (mg/dL)	GH, μg/L (ng/mL)	Cortisol, nmol/L (μg/dL)
Basal	4.7 (85)	0.18 (0.18)	9 (0.33)
30 minutes	1.8 (32)	0.10 (0.10)	11 (0.40)
60 minutes	3.1 (56)	<0.05 (<0.05)	12 (0.43)
90 minutes	6.5 (117)	<0.05 (<0.05)	11 (0.40)
120 minutes	7.8 (141)	0.06 (0.06)	9 (0.33)

Hypothalamic hyperphagia developed, requiring strict diet and lifestyle measures to prevent weight gain, as well as addition of a glucagon-like peptide 1 (GLP-1) agonist. Temperature dysregulation was also noted. Sleep apnea was diagnosed, requiring overnight continuous positive airway pressure.

## Outcome and Follow-up

Nine years after initial presentation, our patient has almost regained full independence in daily activities. However, subtle cognitive deficits of frontosubcortical function remain; in particular, there is slowing of information processing and executive dysfunction, with impaired attention and satiety as well as altered social cognition. He requires lifelong desmopressin and anterior pituitary hormone replacement.

Current treatment regimen consists of:

- desmopressin 120 mcg morning, 120 mcg midday, 240 mcg late afternoon
- hydrocortisone 10 mg twice daily
- thyroxine 200 mcg 4 days/wk, 250 mcg 3 days/wk
- Testogel 2× 5g sachets once daily
- somatropin 0.5 mcg subcutaneously once daily
- liraglutide 1.8 mg once daily
- melatonin 3 mg at night

He requires regular prompting and reminders to take his medications. Occasional inadvertent noncompliance with desmopressin has resulted in hospitalization with hypernatremia. He has also been admitted with hyperpyrexia.

Fertility has been an issue, compounded by ongoing issues with reduced libido and ejaculatory dysfunction. Human chorionic gonadotrophin was trialed, but this was discontinued because our patient did not tolerate cessation of testosterone therapy. Fertility was ultimately achieved through sperm donation.

## Discussion

This case highlights the potentially significant endocrine consequences of HSVEs because of hypothalamic dysfunction. HSV most commonly affects the temporal lobes or frontal lobes. Here, HSV showed a rare predilection for the infundibulum, hypothalamus, subthalamic areas, and optic radiation. This resulted in a clinical complex of visual hallucinations and hyperpyrexia with ensuing endocrine abnormalities. Initial computed tomography brain imaging and CSF cell counts were normal, in keeping with the increasing recognition that more than half of cases of HSVE present with a more slowly evolving picture than clinicians may expect.

Diencephalic dysfunction is rare in herpes simplex encephalitis. As discussed previously, there are few case reports of hypothalamic/pituitary dysfunction in HSVE [3, 4]. The fever at presentation, the serial positive HSV PCRs, and the progression on imaging led us to believe that the diencephalic involvement was direct viral invasion; however, it is possible that osmotic demyelination also played a role in the brain injury. Our patient developed central diabetes insipidus, which led to endocrinology referral and a diagnosis of panhypopituitarism. Diabetes insipidus has previously been reported in herpes zoster encephalitis in an immunocompromised patient with diffuse large-cell lymphoma [5]. Our patient demonstrated dramatic variations in sodium levels. A triphasic response can be seen in neurosurgical patients where initial diabetes insipidus is followed by syndrome of inappropriate antidiuretic hormone secretion (SIADH) and then permanent diabetes insipidus [6]. The underlying mechanism of transient SIADH is thought to be secondary to prestored arginine vasopressin (AVP) being released from dying AVP-producing neurons causing SIADH followed by diabetes insipidus resulting from AVP deficiency when these neurons lose their capacity to synthesize or release AVP. We did not have adequate data in our case to support a diagnosis of SIADH initially, and polydipsia was likely a significant contributor to hyponatremia. Salt and water homeostasis can be complex to manage in those with cerebral injury. This has been outlined by Hannon et al [6] in the neurosurgical patient cohort and highlights the importance of vigilant sodium and fluid balance monitoring in these cases.

Obesity, sleep disorders, and disorders of temperature regulation are recognized features of hypothalamic dysfunction [7]. Obesity can be as a result of hyperphagia, autonomic dysfunction leading to hyperinsulinemia and impaired energy expenditure, and somnolence as a result of disordered sleep [8]. As outlined in the Endocrine Society Clinical Practice Guideline “Evaluation and Treatment of Adult Growth Hormone Deficiency,” GH replacement offers significant benefit in body composition, with studies showing a decrease in total body fat content in response to GH therapy [9]. Our patient was started on GH replacement following flat GH response after insulin tolerance testing. We also commenced GLP-1 agonist therapy in our patient to aid in weight management. There are limited studies examining the efficacy of GLP-1 agonists in hypothalamic dysfunction. Zoicas et al published a case series of 9 patients with hypothalamic obesity. Eight of these 9 patients experienced substantial weight loss (−13.1 kg ± 5.1 kg) with GLP-1 agonist therapy [10].

Our case is unique in that it illustrates the development of panhypopituitarism resulting from hypothalamic dysfunction from HSVE. Further research is needed into the hypothalamic aspects of the neuroendocrine axis. Our case also highlights the longer term difficulty of managing hypothalamic-pituitary dysfunction despite recovery from the acute critical illness.

## Learning Points

- Hypothalamic-pituitary dysfunction is a rare complication of HSV diencephalitis
- Initial computed tomography of the brain and CSF white cell count can be normal in HSV encephalitis
- Salt and water homeostasis can be difficult to manage in those with cerebral injury and require vigilant monitoring of biochemistry, fluid intake, and urine output
- Hypothalamic-pituitary dysfunction can be difficult to manage in the long term because of both the complexities of hypothalamic dysfunction and the need for cautious management of diabetes insipidus and panhypopituitarism, with strict adherence to hormone replacement.

## Data Availability

Data sharing is not applicable to this article because no datasets were generated or analyzed during the current study.

## Abbreviations

AVP: arginine vasopressin
CSF: cerebrospinal fluid
HSV: herpes simplex virus
HSVE: herpes simplex virus encephalitis
GLP-1: glucagon-like peptide
PCR: polymerase chain reaction
SIADH: syndrome of inappropriate antidiuretic hormone secretion
